# Traumatic aortic injury: Computed tomography angiography imaging and findings revisited in patients surviving major thoracic aorta injuries

**DOI:** 10.4102/sajr.v25i1.2044

**Published:** 2021-03-12

**Authors:** Richard Edwards, Nausheen Khan

**Affiliations:** 1 Department of Diagnostic and Interventional Radiology, Faculty of Radiology, University of Pretoria, Pretoria, South Africa; 2 Department of Diagnostic Radiology and Imaging, Faculty of Radiology, University of Pretoria, Pretoria, South Africa

**Keywords:** TAI, traumatic aortic injury, computed tomography angiography, major chest trauma, thoracic aortic injuries

## Abstract

Blunt chest trauma related acute thoracic aortic injury (TAI) is a life-threatening condition that requires prompt diagnosis and appropriate management because of high mortality. Computed tomography angiography (CTA) is the imaging of choice for evaluation of patients with major chest trauma findings suspicious of TAI on chest radiography. This case series describes the CTA findings in four high-velocity incident survivors with associated TAIs, discusses the injury type and treatment, and reviews the literature.

## Introduction

Acute thoracic aortic injury (TAI) is a sequela of blunt thoracic injury with a high mortality rate of 90% if left untreated.^1^ Multi-detector computed tomography (MDCT) with a sensitivity of 98% and a negative predictive value of 100% is the imaging modality of choice for evaluating TAI.

The site of attachment of the thoracic aorta determines the severity of rupture, with the isthmus being involved in 90% of cases because of its immobile position, fixed by the ligamentum arteriosum, a congenital remnant of the ductus arteriosum.^2^ Ascending aortic lesions are rarely seen (5% – 8%), but may be complicated by involvement of the aortic valve resulting in haemopericardium. The descending thoracic aorta is only involved in 1% – 12% of cases.^2^

As mentioned earlier, most vascular injuries sustained to the thoracic aorta are fatal. This case series reveals the findings in four survivors of high-velocity incidents with associated TAIs, including a review of the type of injuries, computed tomography angiography (CTA) findings and a review of the literature.

## Case presentations

Four male patients, aged between 22 years and 45 years, presented to the emergency unit of our hospital, and were suspected of having acute aortic injury on the basis of a widened mediastinum on chest radiography. There was a history of two of them being involved in a motor vehicle accident (MVA), one in a pedestrian vehicle accident (PVA), whilst the fourth patient fell from the fourth floor.

**FIGURE 1:**
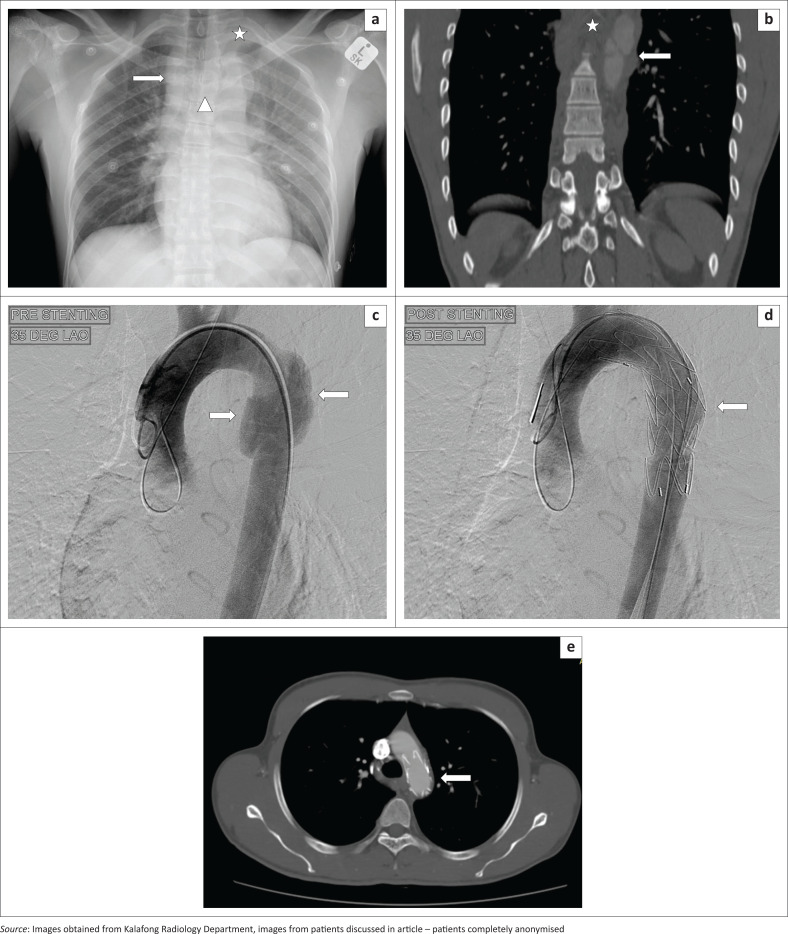
(a) Chest radiography in a 36-year-old male patient involved in a motor vehicle accident. There is widening of the mediastinum (arrow) with a left apical pleural cap (star) and veiling of the left lung field in keeping with a left haemothorax. There is depression of the left main bronchus (triangle), and the silhouette of the aortic knuckle is maintained. (b) Computed tomography angiography demonstrating a mediastinal haematoma (star) and a type 2 aortic injury with an intimal flap and a pseudoaneurysm (arrow). (c) Digital subtraction angiogram image prior to stenting delineating the false aneurysm (arrows) beyond the origin of the left subclavian artery. (d) Digital subtraction angiogram image post successful endovascular aneurysm repair with the stent in place (arrow). (e) Follow-up computed tomography angiography indicates the stent in place with no complications (arrow).

Signs of mediastinal haemorrhage on chest radiography were observed in the first patient (Figure 1a). Computed tomography angiography was proposed by the emergency physician, and it clearly confirmed the site of traumatic aortic injury at the region of aortic isthmus, a Grade 2 injury of the aorta with formation of a false aneurysm (Figure 1b). The digital subtraction angiogram (DSA) confirmed the presence of a false aneurysm, which was treated surgically with endovascular aneurysm repair (EVAR) (Figure 1c & 1d). The patient recovered completely, and a follow-up CTA (Figure 1e) after 6 months revealed no complications.

**FIGURE 2:**
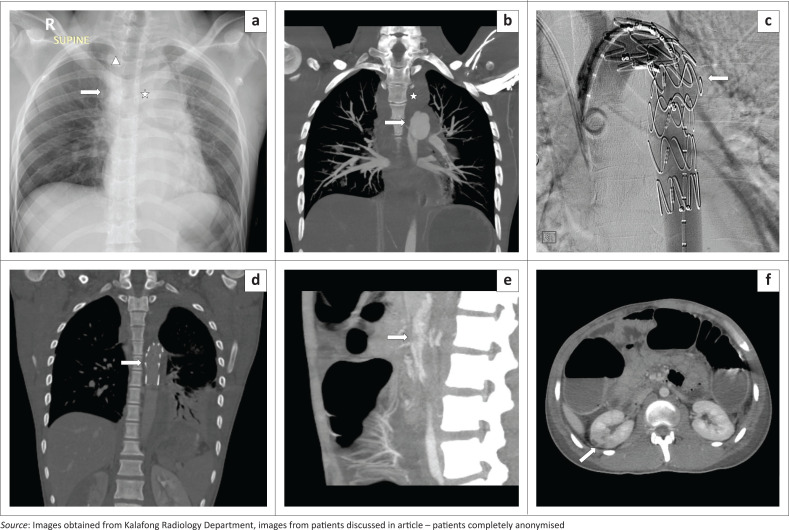
(a) Chest radiography in a 23-year-old male involved in a pedestrian vehicle accident indicates a widened mediastinum (arrow), loss of the aortic knuckle (star), retro-cardiac double density and a widened right paraspinal line (triangle). (b) Coronal computed tomography angiography image with a type 2 aortic injury, complicated by thrombus formation (arrow). Also note the large mediastinal haematoma (star). (c) Sagittal digital subtraction angiogram after endovascular aneurysm repair with the stent in place (arrow). (d) Follow-up computed tomography angiography post stenting. (e) Sagittal computed tomography angiography abdomen demonstrating subtle thrombus within the coeliac artery (arrow). (f) Segmental infarction revealed within the right kidney (arrow).

Case 2 relates to a patient involved in a PVA, with multiple other injuries, including intra-cranial haemorrhage and lower limb fractures. The initial chest radiograph revealed signs of aortic injury (Figure 2a), which was confirmed by CTA as a Grade 2 injury at the aortic isthmus (Figure 2b), followed by successful DSA and EVAR (Figure 2c & 2d). The patient, however, later developed complications secondary to emboli from the EVAR site with peripheral thrombosis in the coeliac artery and its distribution and an infarction in the right kidney, from which he partially recovered (Figure 2e & 2f).

**FIGURE 3:**
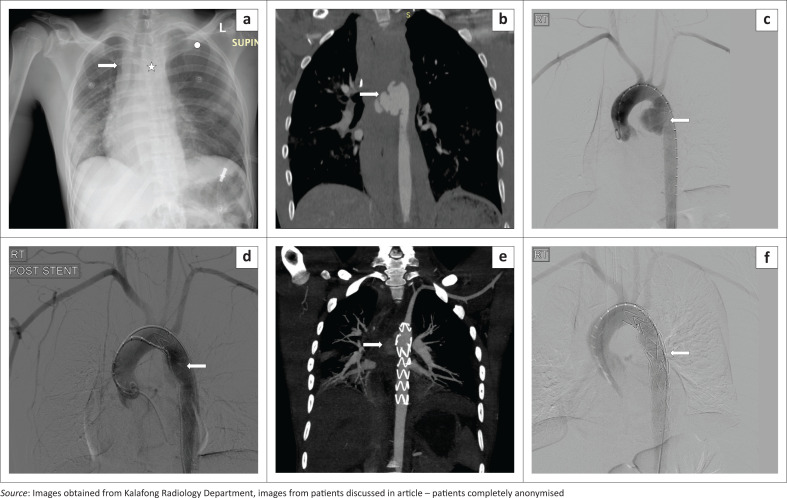
(a) Chest radiography in a 22-year-old male involved in a motor vehicle accident as a front passenger with a widened right paraspinal line (arrow), widened mediastinum (star) and depression of the left main bronchus, all signs pointing towards a vascular injury. Also note the tension pneumothorax on the left (circle) with contralateral mediastinal shift. (b) Computed tomography angiography in the same patient indicating the aortic injury and false aneurysm (arrow) at the isthmus. (c) Initial digital subtraction angiogram image pre-stenting revealing the pseudoaneurysm (arrow). (d) Digital subtraction angiogram image with the stent in place (arrow). (e) Follow-up computed tomography angiography demonstrating a leak around the stent (arrow). (f) A second endovascular stent was placed to treat the endoleak, sagittal oblique digital subtraction angiogram image with double stents (arrow).

The third patient presented with a type 2 aortic injury (Figure 3 a-d), developed an endoleak after EVAR and had to be re-stented (Figure 3e & 3f).

**FIGURE 4:**
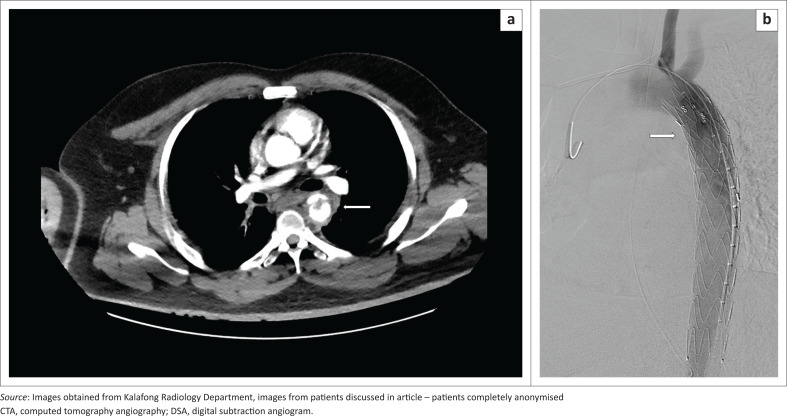
(a) A 45-year-old male jumped from the fourth floor and sustained multiple injuries. Initial computed tomography angiography revealed a traumatic type 2 aortic injury with an intimal flap, thrombus and false aneurysm (arrow). (b) Sagittal oblique digital subtraction angiogram image with the stent in place (arrow). No complications seen.

The fourth patient fell from the fourth floor and sustained multiple additional injuries. He was stented for a type 2 aortic injury (Figure 4a & 4b), which was complicated by a leak, and he required stent repositioning and balloon angioplasty. Complications were not observed at follow-up CTA after the repositioninig, not included within this case series.

This case series describes three different types of blunt chest trauma (MVA, PVA and a fall from height) complicated by type 2 traumatic aortic injury, an area amenable to surgical or endovascular treatment. Because of the unstable condition of the patients and the complexities of additional injuries, EVAR was considered as a therapeutic option. Although two of the cases in this case series developed endoleak complications and one further reported complications of local and distal thrombosis, all four patients recovered completely.

## Discussion

Thoracic aortic injury secondary to blunt chest trauma is a life-threatening emergency with a very high morbidity and mortality rate, which requires urgent medical attention and management. In missed or untreated cases, the mortality has been estimated to be 80% – 90%.^1^

**FIGURE 5:**
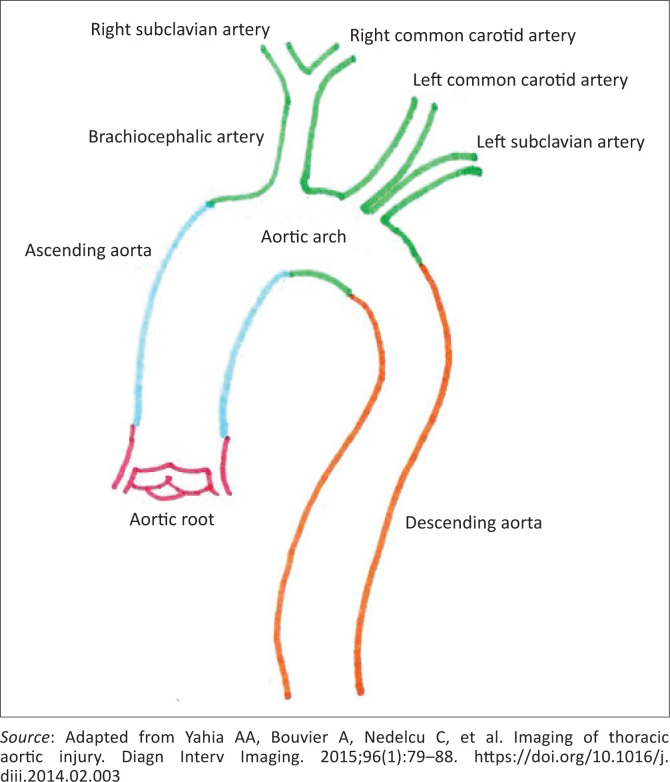
Annotated diagram of different aortic segments. Aortic root (0 in red), segment 1 in blue, segment 2 in green and segment 3 in orange. Isthmus represents the area at the orange and green junction.

The thoracic aorta is anatomically divided into four segments (Figure 5).^2^ The first two portions are intra-pericardial; hence, their involvement results in haemopericardium.^2^

There are several mechanisms that act either in isolation or in combination in TAI, one of the main mechanisms being deceleration with displacement of the heart and displacement, torsion and shearing forces acting on the immobile aorta. Other proposed methods include associated osseous pinch and hydrostatic forces, also known as the water-hammer phenomenon.^2,3,4^

Rapid deceleration including the associated shearing forces exerted, particularly within the frontal and lateral direction, affects front seat passengers in high-velocity MVAs, resulting in injury to the relatively immobile aortic isthmus (ligamentum arteriosum), aortic root and aorta at the region of the diaphragm, contributing to the majority of cases reported.^3^ Ninety per cent of the aortic injuries have shown the involvement of the isthmus.^1,2^

Crass et al.^4^ proposed the theory regarding the ‘osseous pinch’ based on the compression of the aorta between the spine and manubrium, first rib, and/or the medial aspects of the clavicles. The water-hammer effect is secondary to a sudden increase in intra-thoracic pressure that results in a sudden increase in intra-aortic pressure with subsequent rupture of the pericardium and cardiac tamponade.^1,3^

The survival of these patients mainly depends on the site of rupture; the extent of the rupture did not have any bearing on survival. A periaortic haematoma contained by other mediastinal structures and development of a false aneurysm may prolong survival from months to years. Only 2% of the patients live to develop a chronic false aneurysm.^1,5^

Initial imaging in these patients at our institution is a trauma series of radiographs, which includes a chest, abdominal and pelvic radiograph. Chest radiographic signs of TAI have low specificity and include the following: mediastinal widening > 8 cm, abnormal aortic contour, shift of the endotracheal tube and/or trachea to the right, depression of the left or right main stem bronchus, deviation of the nasogastric tube to the right, presence of an apical pleural cap, first rib fracture, acute left-sided haemothorax, retro-cardiac density, loss of the aorto-pulmonary window, widening of the right paraspinal interface, right paratracheal stripe (> 4 mm) and a pneumothorax.^5,6^

Furthermore, if clinically indicated in a polytrauma patient, the emergency physician usually requests a non-enhanced cerebral and cervical computed tomography (CT) scan together with a thoraco-abdominal and pelvic CT scan in the arterial phase followed by an abdominal-pelvic CT scan in the portal phase after injection of 100 (mL) of iodinated contrast agent (Omnipaque 300) at a rate of 4 mL/s – 5 mL/s. Currently, scans at our institute are acquired with a 128-slice Phillips Ingenuity CT scanner and multiplanar reconstruction (MPR), maximum intensity projection (MIP), curved MPR and volume rendering (VR) 3D reconstruction are also used to analyse the available imaging.

The CTA findings of acute TAI have been categorised into direct and indirect or associated findings. Direct findings include intramural haematoma formation, an intimal flap as well as pseudoaneurysmal formation. Indirect or associated findings include periaortic haematoma formation, change in the normal aortic calibre and normal aortic contour.^1,2,3,6,7,8^ In the case of equivocal findings, it becomes a diagnostic challenge for the radiologist if the direct and indirect signs of TAI are absent; in such cases, direct catheter angiography is warranted. Periaortic haematoma in the absence of associated vascular injury may represent injury to the aortic adventitia.^7^

**FIGURE 6:**
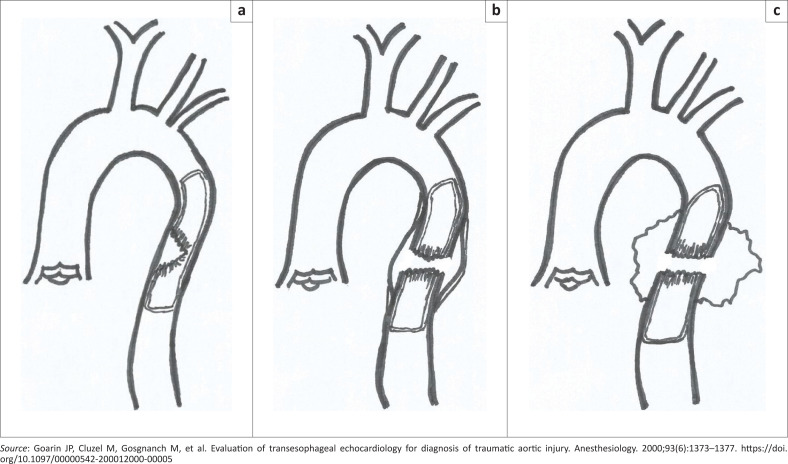
Drawing of aortic injury grading findings at computed tomography angiography: (a) Grade 1: isolated intramural haematoma, (b) Grade 2: involvement of the intima and media, and (c) Grade 3: involvement of intima, media and adventitia.

In 1958, Parmley et al.^1^ pathologically classified traumatic aortic injuries into six groups: (1) intimal haemorrhage, (2) intimal haemorrhage with laceration, (3) medial laceration, (4) complete transection, (5) pseudoaneurysmal formation and (6) periaortic haemorrhage. Heiberg et al.^9^ were the first to describe the appearance of traumatic aortic injury on CT in 1983. Goarin et al.^10^ later classified traumatic aortic lesions into three groups (Figure 6) based on the CTA findings.

Grade 2 lesions, in which both the intima and media are involved, define injuries that include pseudoaneurysm formation and haemomediastinum. Grade 3 lesions are fatal involving all three layers of the tunicae with rapid extravasation of blood.^10^ All of the patients in this case series sustained Grade 2 injuries and survived.

Thoracic aortic injury is uncommon in the paediatric population and accounts for only 2% of paediatric deaths from trauma. This is because of the increased compliance of the chest wall in children, elasticity of the tissues, decreased body mass and lack of atherosclerotic disease.^11^

The pitfalls of CTA in trauma patients include artefacts related to patient movement and breathing, cardiac motion and aortic pulsation, partial volume averaging and support devices. These may cause difficulty in the interpretation of findings in some cases.^2,3,12^ Aortic pulsations may simulate a dissection, which can be avoided by performing a cardiac-gated study.^2,3^ The current problem with routine cardiac gating in cases of chest trauma is the presence of ‘band artefacts’ in patients with a heart rate of over 80 beats per minute, which distorts the findings on MPR and VR images. In the case of ascending aortic pulsatile artefact, a second gated scan after a routine trauma CT chest with 60 mL of intravenous contrast can be performed.

Axial scans are best to exclude ascending aortic injury.^13^ Anatomical variants that a reporting radiologist should be aware of include a ‘ductal remnant’ located at the inferior surface of aorta near the isthmus, as well as the ‘infundibulum’, which is a cone-shaped lesion found at the origin of bronchial or intercostal arteries.^2,3^

Treatment options include three categories, namely, open surgical repair, endovascular repair and medical management. Although open repair of injuries to the aortic root, ascending aorta and aortic arch remains the procedure of choice in recent times, injuries to the aortic isthmus, descending and abdominal aorta have shown successful outcomes with EVAR.^2,3,6^ Advantages of EVAR include a lower mortality rate, a lower risk of paraplegia and no need for invasive open thoracic surgery. However, disadvantages include regular imaging follow-up to asses for graft complications, which include graft migration, graft fracture, endoleak, graft infection and complications related to vascular access.^2,3,6,14^ The patients described in this case series underwent successful endovascular repair with an uneventful follow-up.

Medical management with frequent imaging follow-up is regarded as safe in cases with minimal aortic injuries, defined as intimal flaps measuring < 1 cm with no or minimal periaortic haematoma formation.^15^ Chronic untreated false aneurysms eventually undergo extensive dense calcification. Densely calcified, pain-free aneurysms detected years after the initial trauma have a stable course and may not require surgical intervention.^16^

## Conclusion

Thoracic aortic injury is a serious complication with devastating consequences in untreated patients. A high index of suspicion and careful evaluation are needed for accurate diagnosis of TAI. Computed tomography angiography is the gold standard for diagnosis and -post-treatment monitoring. CT with its high accuracy, negative predictive value, and multiplanar and volumetric abilities can clearly display the aortic and associated injuries and any anatomical variations that may simulate pathology.
